# Prediction of Drug Indications Based on Chemical Interactions and Chemical Similarities

**DOI:** 10.1155/2015/584546

**Published:** 2015-03-02

**Authors:** Guohua Huang, Yin Lu, Changhong Lu, Mingyue Zheng, Yu-Dong Cai

**Affiliations:** ^1^Institute of Systems Biology, Shanghai University, Shanghai 200444, China; ^2^Department of Mathematics, Shaoyang University, Shaoyang, Hunan 422000, China; ^3^State Key Laboratory of Drug Research, Shanghai Institute of Materia Medica, Chinese Academy of Sciences, Shanghai 201203, China; ^4^Department of Mathematics, East China Normal University, Shanghai 200241, China

## Abstract

Discovering potential indications of novel or approved drugs is a key step in drug development. Previous computational approaches could be categorized into disease-centric and drug-centric based on the starting point of the issues or small-scaled application and large-scale application according to the diversity of the datasets. Here, a classifier has been constructed to predict the indications of a drug based on the assumption that interactive/associated drugs or drugs with similar structures are more likely to target the same diseases using a large drug indication dataset. To examine the classifier, it was conducted on a dataset with 1,573 drugs retrieved from Comprehensive Medicinal Chemistry database for five times, evaluated by 5-fold cross-validation, yielding five 1st order prediction accuracies that were all approximately 51.48%. Meanwhile, the model yielded an accuracy rate of 50.00% for the 1st order prediction by independent test on a dataset with 32 other drugs in which drug repositioning has been confirmed. Interestingly, some clinically repurposed drug indications that were not included in the datasets are successfully identified by our method. These results suggest that our method may become a useful tool to associate novel molecules with new indications or alternative indications with existing drugs.

## 1. Background

The biopharmaceutical industry has a problem: its output has not kept pace with the enormous increases in pharmaceutical R&D spending [1]. After nearly two decades of focusing on developing highly selective ligands, the clinical attrition figures challenge the hypothesis “one gene, one drug, one disease” [2]. In addition, there has been a significant investment by pharmaceutical companies on the optimization of drug discovery pipeline using advanced techniques such as structure-based drug design, combinatorial chemistry, HTS, and genomics. However, the impact of these techniques does not change the predicament [3]. Computational approaches may play significant roles in reducing the developmental costs and shortening the paths to approval, for example, to facilitate drug repositioning.

Drug repositioning is “the process of finding new uses outside the scope of the original medical indications for existing drugs or compounds” [4]. In modern computational biology, there are two general approaches to drug repositioning: discovering new indications for an existing drug (drug-centric) and identifying effective drugs for a disease (disease-centric) [5]. The former hypothesizes that “similar drugs” have the same therapeutic effects and are equally effective for a disease, whereas the latter assumes that “similar diseases” need the same therapies and can thus be treated with the same drugs. Different computational approaches related to the drug repositioning problem have been proposed, ranging from clustering drugs either based on their pharmacophore descriptors [6] or based on connectivity map-based networks [7] to predicting drug-target interactions [8–10] and drug-disease associations [11–15].

On the other hand, drug repositioning by computational approaches can be classified into small-scaled applications which analyze specific classes of drugs or drugs for specific diseases [6, 13, 14] and large-scale applications which analyze a relatively large number of drugs and diseases [7, 11, 12, 15, 16]. The datasets vary among different research subjects. Generally, the drugs can be derived from Drugbank [11, 12] or KEGG [17] or FDA approved and practiced drug [15]; the drug indications may originate from the Online Mendelian Inheritance in Man (OMIM) database [11], Drugbank therapeutic categories [12], or DRUGEX system [15]. For the methods allowing large-scale indication predictions, transcriptional responses towards drugs were typically utilized to calculate drug-drug similarity, then the connectivity map was constructed for clustering, and the categories of query drugs were determined by the nearest distance to the clustered communities [7]. Similarly, the integration of the chemical, bimolecular, and clinical information was made to design a general framework based on bipartite network projections, and the drug ranking was calculated by kernelized score functions [12]. From the view of disease pairs, a network-based and guilt-by-association method was applied to predict novel drug indication [15]. In addition to network methods, a logistic regression classifier was built from the classification features originating from drug-drug similarity and disease-disease similarity [11].

In this study, we presented an approach for large-scale identification of drug indications based on a large drug-indication library and the information of chemical interactions in STITCH [18] and chemical similarities in structure. For a given drug, a K-Nearest Neighbor (KNN) ranking strategy was used to predict the indications according to its interactive drugs or similar drugs, based on the assumption that interactive chemicals or similar chemicals in structure are more likely to share similar biological functions [16, 19, 20]. An important merit of the method is that, given a query drug, it can provide a series of candidate indications, ranging from the most likely one to the least likely one. Obviously, the quality and the size of the datasets play a significant role in the predictive ability of a model. We constructed the benchmark dataset from a commercial database, Comprehensive Medicinal Chemistry (CMC) database of Accelrys company [21] that is derived from the Drug Compendium in Pergamon's Comprehensive Medicinal Chemistry, which contains 1,573 drug compounds and 56 indications. The size of dataset in our method is larger than those investigated in most of previous approaches [7, 11, 12]. The performance of the method on this dataset suggests that it can identify the potential disease indications of a query drug.

## 2. Methods

### 2.1. Materials

#### 2.1.1. Dataset

Altogether, 1,944 drug compounds and their indications were retrieved from CMC database. By collecting indications of these drugs, 231 indications recorded in CMC database were obtained. Accordingly, 231 categories were used to label these 1,944 drugs. To yield statistically meaningful result, the categories containing less than 8 drug compounds were disregarded, 1,733 drugs were obtained, and then indications were refined to avoid any inclusion relation between two indications by manual adjustment of the medical terminology mainly based on ATC classification system (http://www.whocc.no/atc_ddd_index/), thereby obtaining 56 categories of indications. For formulation, let DS_1_ denote a dataset consisting of these drugs, and the codes of these drugs and their indications were available in Supplementary Material I (see Supplementary Material available online at http://dx.doi.org/10.1155/2015/584546).

In addition, since some drugs whose structures are very similar may be derived from the same drug, these drugs can be easily correctly predicted by any proper method. To strictly examine the proposed method, these similar drugs should be excluded. For this purpose, a graph was constructed, where nodes represented drugs and two nodes were adjacent if and only if the similarity score of the corresponding drugs based on fingerprint ECFP_4 was at least 0.7 (the reason to select ECFP_4 is explained in Section 3.1). A maximal independent set of 1,573 nodes was extracted from this graph and the corresponding 1,573 drugs in this independent set comprised the dataset DS_2_. These 1,573 drugs were also classified into 56 categories and the similarity score of any two drugs was less than 0.7. Shown in column 3 of Supplementary Material II is the number of drug compounds in each category for dataset DS_2_. For convenience, we used tags *D*_1_, *D*_2_,…, *D*_56_ to represent 56 kinds of indication, where *D*_1_ represented “Antihypertensive,” *D*_2_ “Uterine stimulant,” and so forth (see columns 1 and 2 of Supplementary Material II for details). Accordingly, the dataset DS_2_ can be formulated as follows:

(1) $$\begin{array}{c} \text{D}{\text{S}}_{1}=S_{1}\cup S_{2}\cup \cdots \cup S_{56}, \end{array}$$

where *S*_*i*_ is a subset of DS_2_ containing drugs labeled by indication *D*_*i*_. The detailed codes of drug compounds in each *S*_*i*_ are available in Supplementary Material III.

**Figure 1:**
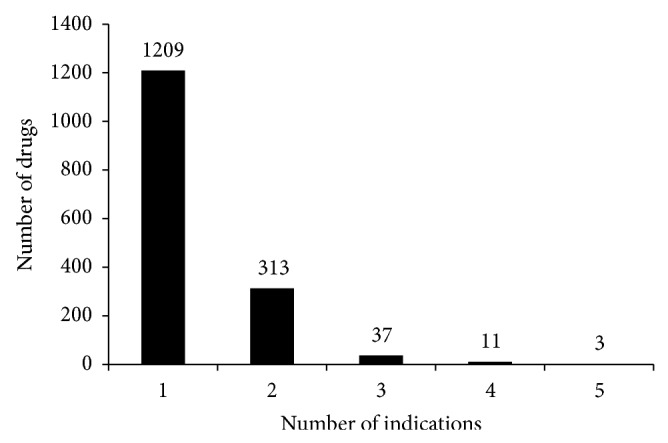
A plot of the number of drugs in DS_2_ versus the number of indications.

It is observed from the last row of Supplementary Material II that the sum of the number of drug compounds in each category is 2,005, which is much larger than 1,573 that is the total number of individual drug compounds investigated in this study, indicating that some drug compounds possess more than one indication; that is, they are present in more than one category. Of the 1,573 drug samples, 1,209 drugs have only one kind of indication, 313 drugs have two kinds of indications, while the rest possess more than two kinds of indications.  Figure 1 shows the relationship between the number of drugs and the number of their corresponding indications. Like the cases of dealing with multilabel classification problems such as predicting multiple attributes of protein or compounds [16, 22, 23], the proposed method would provide the prediction results by ranking the candidate indications from the most likely one to the least one.

In addition, to evaluate the generalization of the proposed method, we employed an independent validation test dataset, denoted by DS_te_, consisting of 32 drug compounds that were gathered from the recently published literature [1, 24, 25]. The drugs in the test dataset meet the following two criteria: (1) involving drug repositioning that has been experimentally confirmed; (2) being not included in DS_1_. These 32 drug compounds and their original indication and reported indication are listed in Table 1.

#### 2.1.2. Chemical Interactions

Some recent studies indicate that interactive compounds are more likely to share common functions than noninteractive ones [16, 26]. The functions of a drug compound can in part determine which diseases it can treat. In view of this, it may be feasible to utilize the information of interactive compounds to predict diseases that a query drug can treat. The information of interactive compounds was downloaded from STITCH (chemical_chemical.links.detailed.v3.1.tsv.gz,  http://stitch.embl.de/) [18], a well-known database containing the interaction information of chemicals and proteins. In detail, chemicals are associated with other chemicals and proteins by evidence derived from experiments, databases, and the literature (http://stitch.embl.de/) in STITCH. In the obtained file, each interaction contains two compounds and five scores that indicate the likelihood of the interaction in five different ways. In detail, the score titled “Similarity” was the Tanimoto 2D chemical similarity score [27, 28] calculated by the open-source Chemistry Development Kit [29]; the score titled “Experimental” was obtained by chemical's activities from MeSH pharmacological actions and NCI60 screens; the score titled “Database” was obtained according to chemical reactions contained in pathway databases; the score titled “Textmining” was obtained based on a cooccurrence scheme and a natural language processing (NLP) approach [30, 31]; while the score titled “Combined_score” integrates all the aforementioned items. For detailed description, readers can refer to Kuhn et al.'s paper [18]. Accordingly, “Combined_score” was used to quantify the interactivity of two compounds: two compounds with the “Combined_score” greater than zero are deemed as interactive compounds. Furthermore, each interaction is labeled by this score, also termed as confidence score in this study, to indicate the likelihood of its occurrence; that is, an interaction with higher confidence score means that the corresponding compounds can interact with each other with higher probability. For two drug compounds *d*_1_ and *d*_2_, the confidence score of the interaction between them is denoted by *w* ^*i*^(*d*_1_, *d*_2_). In particular, if the interaction between two compounds is not reported in STITCH, its confidence score was set to zero.

#### 2.1.3. Chemical Representation and Similarities

The similarity of two compounds in structure is a classic measurement of the relationship of two compounds. Many representation systems have been established to represent compounds. One of the most well-known systems is SMILES (Simplified Molecular Input Line Entry System) [32], a line notation for representing molecules and reactions using ASCII strings. In this study, we also used this system to represent each drug compound. Furthermore, several fingerprints have been established to calculate the similarity of two chemicals based on their SMILES strings up to now [33–35]. Since different fingerprints may induce different similarity scores of two given chemicals, thereby providing different results [36] for some problems of classification and prediction, we tried fingerprints FP2 [33], MACCS [34], ECFP (ECFP_2, ECFP_4, ECFP_6) [35], and FCFP (FCFP_2, FCFP_4, FCFP_6) [35] in this study to calculate the similarity score of chemicals and attempted to select the best one for the prediction of drug indications. For two drug compounds *d*_1_ and *d*_2_, the similarity scores based on different fingerprints, calculated by Open Babel [33] or RDKit [37], were all denoted by *w* ^*s*^(*d*_1_, *d*_2_), where superscript *s* indicated which type of fingerprint was used to calculate similarity scores.

### 2.2. Prediction Method

It has been confirmed that interactive compounds are more likely to share similar functions than non-interactive ones [16, 23]. On the other hand, it is known that compounds with similar structures often share common functions [20]. Because drug indications can be viewed as drug functions, it is appropriate to use known drug indications to predict drugs with unknown indications.

Supposing that there are *n* drugs in the training set *S*′, say *d*_1_, *d*_2_, …, *d*_*n*_, we need to predict the indications of a query drug *d*_*q*_ based on chemical interactions and chemical similarities as follows.

#### 2.2.1. Prediction Based on Chemical Interactions

As described above, interactive compounds often share similar functions [16, 23], thereby having similar indications with higher probability. For a query drug compound *d*_*q*_ and indication *D*_*j*_, the score that *d*_*q*_ possesses *D*_*j*_ was determined by the *k* drug compounds with tag *D*_*j*_ in the training set *S*′, say *d*_*i*_1__, *d*_*i*_2__,…, *d*_*i*_*k*__, such that the confidence scores of the interactions between them and *d*_*q*_ are the first *k* maximum scores, and was calculated by

(2) $$\begin{array}{c} R^{i}\left(d_{q}⟹D_{j}\right)=\overset{k}{\underset{l=1}{\sum }}w^{i}\left(d_{q},d_{i_{l}}\right),\;j=1,2,\ldots ,56, \end{array}$$

where *k* is a predefined positive integer. It is necessary to point out that (2) is identical to the method in Chen et al.'s study [16] (refer to (6) in Chen et al.'s study [16]) when *k* = 1, while it is same as the method in [38] (refer to (3) in Chen et al.'s study [38]) when *k* is set to *n*, where *n* is the size of the training set.

Obviously, the larger the score *R* ^*i*^(*d*_*q*_⇒*D*_*j*_) is, the more likely that the query drug *d*_*q*_ can treat disease *D*_*j*_. When *R* ^*i*^(*d*_*q*_⇒*D*_*j*_) = 0 for some *j*, it means that the likelihood that the query drug having the indication *D*_*j*_ is zero. Because it is a multilabel classification problem where a drug may possess more than one indication, our method provided a series of candidate indications for any query drug, ranging from the most likely one to the least likely one. For example, if the results of (2) were

(3) Ridq⟹D2≥Ri(dq⟹D6)≥Ri(dq⟹D46)⋯≥Ri(dq⟹D23)>0,

it can be inferred that the most likely indication of the query drug is *D*_2_, followed by *D*_6_, *D*_46_, and so forth. Furthermore, *D*_2_ is called the 1st order prediction, *D*_6_ the 2nd order prediction, and so forth.

Note that the outcomes of (2) might be trivial as follows:

(4) $$\begin{array}{c} R^{i}\left(d_{q}⟹D_{j}\right)=0\;\forall j=1,2,\ldots ,56. \end{array}$$

Under such circumstance, there were no interactive compounds of *d*_*q*_ in the training set and no meaningful result can be obtained by this method. We then use the following method based on chemical similarities in structures for further prediction.

#### 2.2.2. Prediction Based on Chemical Similarities

Likewise, because compounds with similar structures often share common functions [20], chemical similarities were applied to predict drug indications if chemical interactions give no meaningful result. For a query drug *d*_*q*_ and indication *D*_*j*_, *k* drug compounds with tag *D*_*j*_ in the training set *S*′, still say *d*_*i*_1__, *d*_*i*_2__,…, *d*_*i*_*k*__, were selected such that the similarity scores between these drug compounds and *d*_*q*_ are the first *k* maximum scores. Now, we calculated the score that *d*_*q*_ can treat indication *D*_*j*_ as follows:

(5) $$\begin{array}{c} R^{s}\left(d_{q}⟹D_{j}\right)=\overset{k}{\underset{l=1}{\sum }}w^{s}\left(d_{q},d_{i_{l}}\right),\;j=1,2,\ldots ,56, \end{array}$$

where *w* ^*s*^(*d*_*q*_, *d*_*i*_*l*__) was the chemical similarity of *d*_*q*_ and *d*_*i*_*l*__ which may be based on FP2, MACCS, ECFP (ECFP_2, ECFP_4, ECFP_6), or FCFP (FCFP_2, FCFP_4, FCFP_6). The rest procedures were same as those of the method based on chemical interactions. Also, given a query drug, the method will provide a series of candidate indications.

#### 2.2.3. Prediction by Integrating Chemical Interactions and Similarities

By integrating chemical interactions and chemical similarities, the indications of a given drug compound *d*_*q*_ were predicted as follows:

- the method based on chemical interactions (cf. (2)) was first applied to predict the indications;
- if the outcomes of (2) are trivial as indicated by (4), the method based on chemical similarities (cf. (5)) was then used to make further prediction.

### 2.3. Cross-Validation and Accuracy Measurement

#### 2.3.1. Cross-Validation Method

In statistical prediction, subsampling test, jackknife test, and independent test are often used to examine the performance of the constructed classifiers [39]. Among these three methods, jackknife test is deemed to be the least arbitrary and can always provide a unique result for a given dataset and a given prediction model because both the training samples and the test samples are fixed [16]. Therefore, it has been widely used by investigators to evaluate the performance of their classifiers [16, 38, 40–49]. Accordingly, it was also used in this study to optimize parameters in methods based on chemical interactions and chemical similarities and compare the performance of different methods.

Subsampling test [50], also named *k*-fold cross-validation, is another widely used cross-validation method. In this method, the dataset is equally and randomly divided into *k* parts. Samples in each part are used as testing samples in turn and samples in the rest *k* − 1 parts train the prediction method. Thus, each sample is tested exactly once. Compared to jackknife test, *k*-fold cross-validation costs less computing time and provides similar predicted results. It has also been used in many studies [19, 51–55]. Accordingly, it was used here to examine the proposed method where *k* was set to 5, that is, 5-fold cross-validation. In addition, we also used independent test to evaluate the proposed method because an independent validation test dataset DS_te_ was constructed as mentioned in Section 2.1.1.

#### 2.3.2. Accuracy Measurement

As described in Section 2.2, the query drug was assigned a series of candidate indications, ranging from the most likely one to the least one. To evaluate the correctness of the candidate indication, the* i*th order prediction accuracy was calculated by

(6) $$\begin{array}{c} {\text{ACC}}_{i}=\frac{\text{P}{\text{D}}_{i}}{N},\;i=1,2,\ldots ,56, \end{array}$$

where *N* denoted the total number of samples, while PD_*i*_ denoted the number of samples whose *i*th order prediction is correct. For example, when *i* = 1, that is, the 1st order prediction accuracy, the 1st order prediction of each investigated sample was collected and PD_1_ was the number of these predictions which were correct, thereby obtaining the 1st order prediction accuracy according to (6). It is obvious that ACC_*i*_ is the ratio of correct *i*th order predicted samples to all samples. If a prediction method yields high ACC_*i*_ with small *i* and low ACC_*i*_ with large *i*, it is deemed as an effective prediction. Since it is difficult to infer the number of indications for certain drug, investigators always pay more attention to the 1st order prediction than others. On the other hand, the 1st order prediction of certain drug indicated its most likely indication. In view of this, the first order prediction accuracy is the most important indicator of the performance of the method.

On the other hand, in pattern recognition and information retrieval, recall and precision are often used to evaluate the performance of the method. For multilabel classification problem, recall and precision of the first *t* order predictions can be calculated by the following formulae:

(7) $$\begin{array}{c} \text{Recal}{\text{l}}_{t}=\frac{1}{N}\overset{N}{\underset{j=1}{\sum }}\frac{P_{j}^{t}}{N^{j}},\\ \text{Precisio}{\text{n}}_{t}=\frac{1}{N}\overset{N}{\underset{j=1}{\sum }}\frac{P_{j}^{t}}{t}, \end{array}$$

where *N* ^*j*^ represented the number of known indications of the *j*th sample in the dataset and *P*_*j*_^*t*^ represented the number of correct predictions of the *j*th sample in the dataset among its first *t* order predictions. Obviously, ACC_1_ = Precision_1_. Since different drug compounds have different numbers of known indications, we set the parameter *t* in (7) to the smallest integer that is no less than the average number of known indications in the dataset, which can be computed by

(8) $$\begin{array}{c} \text{Average}=\frac{\sum_{j=1}^{N}N^{j}}{N}; \end{array}$$

that is, *t* = ⌈Average⌉. Obviously, larger Recall_*t*_ and Precision_*t*_ imply better prediction performance of the method.

## 3. Results and Discussion

### 3.1. Optimization of the Methods Based on Chemical Similarities and Chemical Interactions

As mentioned in Section 2.1.3, eight types of fingerprints, including ECFP (ECFP_2, ECFP_4, ECFP_6), FCFP (FCFP_2, FCFP_4, FCFP_6), FP2, and MACCS, were used to calculate the similarity score of two chemicals. To build a more effective prediction method, it is necessary to compare the performance of the method based on chemical similarities on DS_1_, where chemical similarities were calculated based on different types of fingerprints and *k* was set to 1, 2, …, 15, 1732. The performance of these methods evaluated by jackknife test was available as Supplementary Material IV. It can be observed that when the similarity scores were based on same type of fingerprint, the 1st order prediction accuracies followed an increasing trend before reaching the highest accuracy and then followed a descending trend.  Table 2 lists the highest 1st order prediction accuracies for different types of fingerprint and the values of *k* with which these accuracies can be obtained. It is easy to see that using ECFP_4 and setting *k* = 2 provided the highest 1st order prediction accuracy. Thus, we used this type of fingerprint and set *k* = 2 to build the method based on chemical similarities. In addition, since the proposed method integrated the method based on chemical similarities, the similar drug compounds under fingerprint ECFP_4 should be excluded in order to strictly examine our method. In view of this, the similarity scores based on fingerprint ECFP_4 were used to refine the dataset DS_1_ by setting the threshold 0.7, thereby obtaining the dataset DS_2_.

In the dataset DS_2_, there were 896 drug compounds that have the information of chemical interactions. These drugs comprised the dataset DS^(*i*)^. The classification model based on chemical interactions (cf. (2)) was conducted on DS^(*i*)^. To select an optimal parameter *k*, it was evaluated by jackknife test and *k* was set to 1, 2, …, 15, 895. The prediction accuracies thus obtained are available in Supplementary Material V, from which we can observe that the 1st order prediction accuracies followed an increasing trend with the increasing of *k* when *k* < 5, while the accuracies descended with the increase of *k* when *k* > 5 (see Table 3 for details). Since the parameter *k* means the number of interactions that were used to calculate the score that the query drug possesses a certain indication, the score cannot reflect the true likelihood that the query drug has an indication when *k* is small, while with the increase of *k*, more and more interactions with low confidence scores are added, which may be noises to the prediction, thereby influencing the predicted results. The highest 1st order prediction accuracy of 58.48% was obtained when *k* was set to 5. Thus, we set *k* = 5 for the method based on chemical interactions.

### 3.2. Performance of the Proposed Method on DS_2_

For clarity, the dataset DS_2_ is separated into two subsets, DS^(*i*)^ and DS^(*s*)^, where DS^(*i*)^ consisted of 896 drug compounds that have the information of chemical interactions, while DS^(*s*)^ contained the rest 677 drug compounds that have no such information. Then the method based on chemical interactions with *k* = 5 was applied to process DS^(*i*)^, while the method based on chemical similarities with fingerprint ECFP_4 and *k* = 2 was used to process DS^(*s*)^. The predicted results thus obtained are given as follows.

#### 3.2.1. Performance of the Method Based on Chemical Interactions on DS^(*i*)^

Using the 896 drugs in DS^(*i*)^, the classification model based on chemical interactions (cf. (2)) with *k* = 5 was constructed and evaluated by 5-fold cross-validation. To widely examine the method, it was executed five times on DS^(*i*)^. The predicted results thus obtained are available in Supplementary Material VI.  Table 4 lists the first 20 prediction accuracies for each time. It can be seen that the 1st order prediction accuracies were between 55% and 58% and the mean value of these accuracies was 57.00%. For each time, the prediction accuracies generally followed a descending trend with the increase of the order number, indicating that the candidate indications of the samples in DS^(*i*)^ were sorted quite well. In addition, the standard deviations of the five prediction accuracies with the same order were almost lower than 1%, indicating that this method was quite stable on DS^(*i*)^. The average number of indications that samples in DS^(*i*)^ can treat was 1.31; that is, Average = 1.31. Thus, the first two predictions of each sample in DS^(*i*)^ were considered. After calculating (7) with *t* = 2, we obtained 5 Recalls and 5 Precisions, listed in columns 2 and 3 of Table 5. The mean values of Recalls and Precisions were 62.29% and 39.76%, suggesting that the method based on chemical interactions is quite effective to the prediction of drug indications.

#### 3.2.2. Performance of the Method Based on Chemical Similarities on DS^(*s*)^

For the 677 drugs in DS^(*s*)^ that have no information of chemical interactions, the method based on chemical similarities (cf. (5)) with fingerprint ECFP_4 and *k* = 2 was used to make prediction and evaluated by 5-fold cross-validation. Also, this method was executed 5 times. The predicted results thus obtained are also available in Supplementary Material VI (the first 20 prediction accuracies for each time are listed in Table 6), from which we can see that five 1st order prediction accuracies were between 43% and 46%. The mean value of these accuracies was 44.45%. Similarly, the prediction accuracies always followed a descending trend with the increase of prediction order for each time, indicating that the method based on chemical similarities also arranged the candidate indications of the samples in DS^(*s*)^ quite well. It can also be observed from Supplementary Material VI that the standard deviations of the five prediction accuracies with the same order were all lower than 1%, indicating that this method was quite stable on DS^(*s*)^. The average number of indications that drugs in DS^(*s*)^ can treat was 1.22. Thus, we still considered the first two predictions for each sample in DS^(*s*)^ which produced 5 Recalls and 5 Precisions by (7) with *t* = 2. These values are listed in columns 4 and 5 of Table 5, from which we can observe that the mean values of Recalls and Precisions were 48.62% and 28.65%, respectively. These results indicate that the method based on chemical similarities is also effective in the prediction of drug indications.

#### 3.2.3. Performance of the Integrated Method on DS_2_

The integrated method combined the predicted results mentioned in Sections 3.2.1 and 3.2.2. The predicted results for each of 5 times were also available in Supplementary Material VI, while Table 7 lists the first 20 prediction accuracies obtained by the integrated method for each time. It can be seen that the five 1st order prediction accuracies were between 50% and 53% and the mean value of these accuracies was 51.48%. Furthermore, the standard deviations of the five prediction accuracies with the same order were all lower than 1%, suggesting that the integrated method was quite stable on DS_2_. The average number of indications of samples in DS_2_ was 1.27 (2,005/1,573), meaning that the average correct rate would be 1.27/56 = 2.27% if one predicts them by random guess. It is much lower than the five 1st order prediction accuracies obtained by the integrated method. In view of the average number, we consider the first two predictions for each sample in DS_2_. The outcomes of (7) with *t* = 2 yield 5 Recalls and 5 Precisions, which are listed in columns 6 and 7 of Table 5. The mean value of Recall and Precision was 56.28% and 34.87%, respectively.

In addition, to sufficiently indicate the effectiveness of the integrated method, we collected the first two predictions for each sample in DS_2_ and calculated the prediction accuracy for each category *D*_*i*_, which was computed by

(9) $$\begin{array}{c} \text{S}{\text{N}}^{i}=\frac{\text{T}{\text{P}}^{i}}{C^{i}},\;i=1,2,\ldots ,56, \end{array}$$

where *C* ^*i*^ denoted the number of drug compounds labeled by *D*_*i*_, that is, *C* ^*i*^ = |*S*_*i*_|, and TP^*i*^ denoted the number of drug compounds whose 1st order prediction or 2nd order prediction was *D*_*i*_. These accuracies were listed in Supplementary Material VII. It can be seen that the mean values of accuracies of 12 categories were higher than 60%, where 2 of them (*D*_11_, *D*_56_) were higher than 80%. It is known that the category of large size can easily receive high prediction accuracy, while the category of small size can easily receive low prediction accuracy. However, this case should be avoided for an effective prediction method. To evaluate our method in this aspect, that is, investigating the linear correlation between the prediction accuracy of each category and the size of each category, we employed Pearson product-moment correlation coefficient which is a widely used measure of the linear correlation between two variables and can be computed by

(10) $$\begin{array}{c} r=\frac{\sum_{i=1}^{n}\left(x_{i}-\overset{-}{x}\right)\left(y_{i}-\overset{-}{y}\right)}{\sqrt{\sum_{i=1}^{n}{\left(x_{i}-\overset{-}{x}\right)}^{2}\sum_{i=1}^{n}{\left(y_{i}-\overset{-}{y}\right)}^{2}}}, \end{array}$$

where $\overset{-}{x}$ is the mean value of *x*_1_, *x*_2_,…, *x*_*n*_ and $\overset{-}{y}$ is the mean value of *y*_1_, *y*_2_,…, *y*_*n*_. Here, we set *x*_*i*_ to be the mean value of five SN^*i*^, that is, values in the last column of Supplementary Material VII, and set *y*_*i*_ to be the number of drug compounds labeled by *D*_*i*_ divided by 2,005, that is, *y*_*i*_ = |*S*_*i*_ | /2005, where 2,005 was the sum of the number of drug compounds in each category. By (10), the obtained rate was 0.53, yielding that the linear correlation of these two variables was not significant. For example, the categories *D*_56_ and *D*_11_ obtained the highest two prediction accuracies (cf. Supplementary Material VII); however, their sizes were only 7 and 14 (cf. Supplementary Material II) which were very small. All of these results indicate that the integrated method performed quite well for the prediction of drug indications.

### 3.3. Comparison of Different Methods

**Figure 2:**
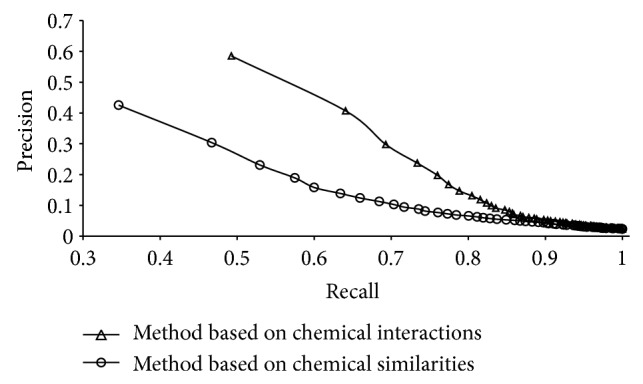
Two curves with Recalls as their *X*-axis and Precisions as their *Y*-axis. Recalls and precisions were obtained by method based on chemical interactions with *k* = 5 and method based on chemical similarities with fingerprint ECFP_4 and *k* = 2.

At a first glance at the Supplementary Material VI, the method based on chemical interactions with *k* = 5 seems to outperform the method based on chemical similarities with fingerprint ECFP_4 and *k* = 2. However, these predicted results were derived from two different datasets. To make a comparison using the same dataset, we executed the method based on chemical similarities with fingerprint ECFP_4 and *k* = 2 on DS^(*i*)^, in which each sample can be predicted by the method based on chemical interactions. It was also evaluated by jackknife test. Listed in columns 2 and 3 of Supplementary Material VIII are the prediction accuracies obtained by the methods for the prediction of indications that samples in DS^(*i*)^ can treat. The 1st order prediction accuracy by the method based on chemical interactions was 58.48%, while it was 42.52% by the method based on chemical similarities. To compare the performance of the methods more thoroughly, we calculated Recall and Precision for the first *t* order predictions and plot two curves with Recalls as their *X*-axis and Precisions as their *Y*-axis.  Figure 2 shows the two curves, from which we can see that the Recall and Precision obtained by the method based on chemical interactions are always higher than those obtained by the method based on chemical similarities. All of these indicate that the method based on chemical interactions is superior to the method based on chemical similarities for the prediction of drug indications. Thus, we arranged the method based on chemical interactions as the first choice while the method based on chemical similarities as a backup. The arrangement in this study conforms to the results in Chen et al.'s study [16]. The main reason is that the confidence score of an interaction between two compounds, which was used in the method based on chemical interactions, contains different kinds of information of compounds, such as their activities, structures, reactions, and so forth [18], while the method based on chemical similarities only used the information of compound structures.

The integrated method proposed in this study sequentially used the confidence scores of interactions between chemicals and similarity scores of chemicals. Another simple integrated scheme, termed as the method based on integrated scores, is to combine these scores in advance and then make prediction. Given a query drug *d*_*q*_, the score that *d*_*q*_ can treat indication *D*_*j*_ was computed by

(11) Rintegrateddq⟹Dj=Ridq⇒Dj+Rsdq⇒Dj2,hhhhhhhhhhhhhhhhhhhhhhhhhhhhhhhj=1,2,…,56,

where *s* is ECFP_4 and the parameters *k* in *R* ^*i*^(*d*_*q*_⇒*D*_*j*_) and *R* ^*s*^(*d*_*q*_⇒*D*_*j*_) were 5 and 2, respectively. The following procedure was same as those of the method based on chemical interactions and chemical similarities.

The original motive of employing this method is to make comparison with the proposed method. However, since *R* ^*i*^(*d*_*q*_⇒*D*_*j*_) = 0 (*j* = 1, 2, …, 56) for each sample in DS^(*s*)^, that is, the predicted results obtained by the method based on chemical similarities and the method based on integrated scores on DS^(*s*)^ were same, the method based on integrated scores was conducted on DS^(*i*)^ evaluated by jackknife test. The obtained prediction accuracies were listed in column 4 of Supplementary Material VIII, from which we can see that the 1st order prediction accuracy was 58.82%. It was almost same as that of the method based on chemical interactions with *k* = 5, while it was much higher than that of the method based on chemical similarities with fingerprint ECEP_4 and *k* = 2. It can be easily inferred that this integrated scheme and the method based on chemical interactions were almost at the same level. Since the confidence score of two chemicals, used in the method based on chemical interactions, contains the information of their similarity information [18], that is, the score calculated by (5) and added to (11) was redundant, it is reasonable that the performance of these two methods was almost the same. It can be further inferred that the performance of the method based on integrated scores and that of the proposed method were also at the same level, because the predicted results obtained by the method based on chemical similarities and the method based on integrated scores on DS^(*s*)^ were the same.

### 3.4. Performance of the Integrated Method on *DS*_*te*_

The integrated method combined the method based on chemical interactions with *k* = 5 and the method based on chemical similarities with fingerprint ECEP_4 and *k* = 2. To test the generalization of this method, it was conducted on DS_te_ to predict indications of drug compounds in it. To calculate the prediction accuracy, the original indications and reported indications of each sample in DS_te_ were combined together as the known indications, thereby yielding the 1st prediction accuracy of 50.00%, which is almost identical to the 1st prediction accuracy obtained by the method on DS_2_. Furthermore, the 2nd prediction accuracy was 21.88%. All of these suggest that the proposed method has a good generalization.

### 3.5. Illustration of the Predictive Results

Since 5-fold cross-validation is unstable, that is, different partitions may produce different predictions for a given sample, the analysis of the results evaluated by 5-fold cross-validation is not very reliable. On the other hand, jackknife test can avoid this case. In view of this, the integrated method was again conducted on DS_2_, evaluated by jackknife test. The obtained prediction accuracies for the methods based on chemical interactions and chemical similarities and integrated method were available as Supplementary Material IX. The 1st order prediction accuracies of the method based on chemical interactions on DS^(*i*)^, the method based on chemical similarities on DS^(*s*)^, and the integrated method on DS_2_ were 58.48%, 47.27%, and 53.66%, respectively, which were a little higher than the corresponding methods on the datasets evaluated by 5-fold cross-validation. In addition, the Recalls of the first two predictions for three methods were 64.08%, 51.38%, and 58.61%, respectively, while the Precisions were 40.68%, 30.21%, and 36.17% for three methods, respectively. In the following paragraphs of this section, further discussions were described based on predictions of each sample in DS_2_ and DS_te_.

Interestingly, some examples in DS_2_ showed that the new clinical indications were predicted in the first 2 order predictive diseases based on chemical similarities. From the jackknife test of the dataset DS_2_ which contains 1,573 drug compounds, we analyzed several examples that new indications were accurately predicted which were not included in the original datasets. We presented the results as follows: thalidomide (CID000005426), whose original indication is antiemetic in pregnancy [56] and new indication is multiple myeloma (acted as TNF-*α* inhibitor) [57], is predicted to treat diseases such as antineoplastic (1st order prediction, new clinical indication) and antibacterial (2nd order prediction); leflunomide (CID000003899), whose original indication is rheumatoid arthritis (targeted at DHODH) [58] and new indication is prostate cancer (targeted at PDGEF, EGFR, FGFR and NF-*κ*B) [59], is predicted to treat disease such as antineoplastic (1st order prediction, new clinical indication) and antiinflammatory (2nd order prediction); chlorpromazine (CID000002726), whose original indication is antiemetic (antihistamine) [60] and new indication is nonsedating tranquillizer (dopamine receptor blockade) [61], is predicted to treat disease such as Anxiolytic (1st order prediction, new clinical indication) and antipsychotic (2nd order prediction).

The indications of samples in DS_te_ were also predicted by our method. As described in Section 3.4, the 1st order prediction accuracy was 16/32 = 50.00% and the 2nd order prediction accuracy was 7/32 = 21.88%. Meanwhile, 20 out of 32 drugs were correctly predicted for the first two orders, where 15 out of 32 drugs were predicted correctly in aspect of original indications and 8 out of 32 drugs were predicted correctly in aspect of repositioned indication, although 3 out of the 8 drugs were predicted correctly responding to the original indication. The description of 8 instances with accurate prediction of new indication in validation test set was shown in Table 8.

Further, some of our predictions are supported by* in vitro* assay results from different sources, which may provide mechanism-based interpretation of these potential novel indications. For example, for Quinacrine (CID000000237), the 2nd ranked indication is antiinflammatory. Several researches [62, 63] indicated that Quinacrine is an inhibitor of cytosolic phospholipase A2, which selectively hydrolyzes arachidonyl phospholipids in the sn-2 position releasing arachidonic acid. Together with the lysophospholipid activity, quinacrine is implicated in the initiation of the inflammatory response. The predicted indication of Colesevelam (CID00000160051) is antidiabetic (2nd indication). As we know, Colesevelam acts as bile acid sequestrants in the gastrointestinal tract upregulate bile acid synthesis (via cholesterol 7-alpha-hydroxylase) by means of utilizing cholesterol and reduced low-density lipoprotein cholesterol levels [64]. Although the exact mechanism of action for the glucose-lowering effect of Colesevelam is still unclear, it may exert the glycemic effect by altering the interaction of the bile acid pathways [65, 66]. From the above two cases, we may find that the prediction of our model may provide useful information for identifying new possible indications of some existing drugs.

These results demonstrated that our method can successfully identify some potential new indications for a drug, which supported the hypothesis that “similar drugs” are more likely to have the same therapeutic effects. In our method, interacted drugs were also considered “similar drugs.”

## 4. Conclusions

In the study, we built an effective classifier to predict drug indications based on chemical interactions extracted from STITCH database and chemical structure similarity. The predictor based on chemical interactions outperformed the predictor based on chemical similarities. Therefore, we arranged chemical interaction before chemical similarity to build the predictor for each drug; that is, if the disease indications of a drug cannot be predicted by chemical interaction, then they are predicted by chemical similarity. As a result, the Recall rate and Precision of the first two predictions are 56.28% and 34.87%, respectively. As to the independent test set, the model yielded the accuracy of 50.00% for the 1st prediction and 21.88% for the 2nd prediction. And interestingly, some drug repositioning instances are correctly implicated by our method. A limitation of the method is that only 56 categories of drug indications are analyzed, which may be improved with the expansion of the drug indication data.

## Supplementary Material

The Supplementary Material contains nine files. In detail, Supplementary Material I lists 1,733 drug compounds in the dataset DS_1_ and their indications; Supplementary Material II lists the number of drug samples in each category for dataset DS_2_; Supplementary Material III lists the detailed samples in each category for dataset DS_2_; Supplementary Material IV lists the performance of the method based on chemical similarities, where similarity scores were computed based on 8 types of fingerprints and k was set to 1, 2,…, 15, 1732; Supplementary Material V lists the prediction accuracies with different k obtained by the method based on chemical interactions on DS^(i)^ evaluated by jackknife test; Supplementary Material VI lists the prediction accuracies obtained by three methods on DS^(i)^, DS^(s)^, DS_2_, evaluated by 5-fold cross-validation; Supplementary Material VII lists the prediction accuracy for each category by collecting the first two predictions obtained by the integrated method on DS_2_; Supplementary Material VIII lists the prediction accuracies obtained by methods based on chemical interactions, chemical similarities and integrated scores on DS^(i)^ evaluated by jackknife test; Supplementary Material IX lists the prediction accuracies obtained by three methods on DS^(i)^, DS^(s)^, DS_2_, evaluated by jackknife test.

## Figures and Tables

**Table 1:** Detailed information of samples in DS_te_.

Name	ID	Original indication	Reported indication
Statins	CID000446156	Myocardial infarction	Prostate cancer, leukemia
Metformin	CID000004091	Diabetes mellitus	Breast cancer, adenocarcinoma, prostate, colorectal cancer
Rapamycin	CID005284616	Immunosuppressant	Colorectal cancer, lymphoma, leukemia
Methotrexate	CID000126941	Acute leukemia	Osteosarcoma, breast cancer, Hodgkin lymphoma
Zoledronic acid	CID000068740	Antibone resorption	Multiple myeloma, prostate cancer, breast cancer
Wortmannin	CID000312145	Antifungal	Leukemia
Thiocolchicoside	CID000072067	Muscle relaxant	Leukemia, multiple myeloma
Noscapine	CID000275196	Antitussive, antimalarial, analgesic	Multiple cancer types
Galantamine	CID000009651	Polio, paralysis, anaesthesia	Alzheimer's disease
Ropinirole	CID000005095	Hypertension	Parkinson's disease, idiopathic restless leg syndrome
Tofisopam	CID000005502	Anxiety-related conditions	Irritable bowel syndrome
Finasteride	CID000057363	Benign prostatic hyperplasia	Hair loss
Mifepristone	CID000055245	Pregnancy termination	Psychotic major depression
Minoxidil	CID000004201	Hypertension	Hair loss
Paclitaxel	CID000036314	Cancer	Restenosis
Phentolamine	CID000005775	Hypertension	Impaired night vision
Sildenafil	CID000005212	Angina	Male erectile dysfunction
Tadalafil	CID000110635	Cardiovascular disease, inflammation	Male erectile dysfunction
Topiramate	CID005284627	Epilepsy	Obesity
Zidovudine	CID000035370	Cancer	HIV/AIDS
Allopurinol	CID000002094	Tumor lysis syndrome	Gout
Amphotericin	CID005280965	Fungal infections	Leishmaniasis
Colchicine	CID000006167	Gout	Recurrent pericarditis
Retinoic acid	CID000444795	Acne	Acute prophylaxis
Bimatoprost	CID005311027	Glaucoma	Promoting eyelash growth
Ceftriaxone	CID005479530	Bacterial infections	Amyotrophic lateral sclerosis
Colesevelam	CID000160051	Hyperlipidemia	Type 2 diabetes mellitus
Disulfiram	CID000003117	Alcoholism	Melanoma
Naproxen	CID000156391	Inflammation, pain	Anti-Alzheimer's disease
Minocycline	CID054675783	Acne	Ovarian cancer, glioma
Dapoxetine	CID000071353	Analgesia, depression	Premature ejaculation
Bromocriptine	CID000031101	Parkinson's disease	Diabetes mellitus

**Table 2:** Best performance of the method based on chemical similarities for different types of fingerprint and values of *k*.

Type of fingerprint	Highest 1st order prediction accuracy (%)	*k*
ECFP_2	48.70	3
ECFP_4	49.39	2
ECFP_6	49.11	5
FCFP_2	42.87	2,3
FCFP_4	48.07	3
FCFP_6	48.99	3
FP2	43.91	3
MACCS	43.39	2,3

**Table 3:** The 1st order prediction accuracies with different *k* obtained by the method based on chemical interactions on DS^(*i*)^ evaluated by jackknife test.

Value of *k*	The 1st order prediction accuracy
1	47.77%
2	55.92%
3	57.59%
4	58.26%
5	58.48%
6	58.37%
7	58.15%
8	58.04%
9	58.04%
10	58.04%
11	57.81%
12	57.81%
13	57.70%
14	57.70%
15	57.70%
895	57.59%

**Table 4:** The first 20 prediction accuracies obtained by the method based on chemical interactions on DS^(*i*)^ evaluated by 5-fold cross-validation for 5 times.

Order	First time (%)	Second time (%)	Third time (%)	Fourth time (%)	Fifth time (%)	Mean (%)	Standard deviation (%)
1	56.37	55.95	57.31	57.47	57.92	57.00	0.82
2	21.98	24.01	22.03	22.17	22.35	22.51	0.85
3	8.91	7.25	8.90	6.90	6.84	7.76	1.06
4	5.98	5.32	4.22	5.77	5.25	5.31	0.68
5	3.16	4.19	4.11	4.41	4.56	4.09	0.55
6	2.59	2.49	2.40	2.04	1.94	2.29	0.29
7	1.47	2.38	2.51	2.49	2.51	2.27	0.45
8	1.69	1.47	1.26	1.36	1.60	1.47	0.18
9	2.37	1.13	1.48	1.36	1.25	1.52	0.49
10	0.68	1.02	1.48	1.02	0.91	1.02	0.29
11	1.24	1.25	0.80	1.24	0.91	1.09	0.22
12	1.01	1.02	1.37	1.13	1.37	1.18	0.18
13	1.35	1.25	1.03	1.24	1.14	1.20	0.12
14	0.90	0.45	0.57	0.68	0.57	0.63	0.17
15	0.56	0.57	0.91	0.79	0.68	0.70	0.15
16	0.68	0.79	0.46	0.23	0.57	0.54	0.22
17	1.13	0.79	0.68	1.24	0.46	0.86	0.32
18	0.90	0.79	0.23	1.13	0.57	0.72	0.34
19	1.13	0.57	0.91	1.02	0.68	0.86	0.23
20	0.56	1.36	1.26	0.68	1.14	1.00	0.36

**Table 5:** The Recalls and Precisions of the first two predictions obtained by three methods on DS^(*i*)^, DS^(*s*)^, and DS_2_, respectively.

Order of time	DS^(*i*)^		DS^(*s*)^		DS_2_	
	Recall (*t* = 2) (%)	Precision (*t* = 2) (%)	Recall (*t* = 2) (%)	Precision (*t* = 2) (%)	Recall (*t* = 2) (%)	Precision (*t* = 2) (%)
1st	61.55	39.18	48.95	28.79	56.06	34.65
2nd	62.37	39.98	47.81	28.26	55.99	34.84
3rd	62.42	39.67	47.24	27.76	55.69	34.39
4th	62.45	39.82	49.68	29.32	56.86	35.22
5th	62.68	40.14	49.39	29.09	56.80	35.25

Mean	62.29	39.76	48.62	28.65	56.28	34.87

**Table 6:** The first 20 prediction accuracies obtained by the method based on chemical similarities on DS^(*s*)^ evaluated by 5-fold cross-validation for 5 times.

Order	First time (%)	Second time (%)	Third time (%)	Fourth time (%)	Fifth time (%)	Mean (%)	Standard deviation (%)
1	44.17	43.62	43.90	45.86	44.68	44.45	0.88
2	13.41	12.90	11.62	12.77	13.51	12.84	0.75
3	6.85	5.94	8.18	6.39	5.89	6.65	0.94
4	5.54	6.67	4.73	5.22	6.90	5.81	0.93
5	4.52	4.06	5.45	3.92	4.45	4.48	0.60
6	2.19	3.33	3.87	4.64	3.02	3.41	0.92
7	3.64	3.33	2.30	2.90	2.73	2.98	0.53
8	1.90	1.74	3.16	1.31	3.16	2.25	0.86
9	2.77	2.75	2.30	2.32	1.58	2.34	0.48
10	2.48	3.48	1.43	1.74	1.44	2.11	0.87
11	2.04	1.45	1.72	1.16	2.44	1.76	0.50
12	2.19	2.61	2.01	2.76	1.44	2.20	0.52
13	2.33	1.74	2.58	2.03	1.29	2.00	0.50
14	2.19	2.17	2.73	1.31	2.30	2.14	0.52
15	0.44	1.88	1.15	1.60	1.58	1.33	0.56
16	1.02	1.16	0.72	1.16	1.15	1.04	0.19
17	0.87	0.87	1.29	1.02	1.15	1.04	0.18
18	0.87	1.16	1.29	1.02	0.72	1.01	0.23
19	1.60	1.01	1.87	1.31	1.01	1.36	0.37
20	1.60	0.72	0.72	1.02	1.29	1.07	0.38

**Table 7:** The first 20 prediction accuracies obtained by the integrated method on DS_2_ evaluated by 5-fold cross-validation for 5 times.

Order	First time (%)	Second time (%)	Third time (%)	Fourth time (%)	Fifth time (%)	Mean (%)	Standard deviation (%)
1	51.05	50.54	51.37	52.38	52.07	51.48	0.75
2	18.25	19.14	17.42	18.05	18.44	18.26	0.62
3	8.01	6.68	8.58	6.68	6.42	7.27	0.96
4	5.79	5.91	4.45	5.53	5.98	5.53	0.63
5	3.75	4.13	4.70	4.20	4.51	4.26	0.37
6	2.42	2.86	3.05	3.18	2.42	2.78	0.36
7	2.42	2.80	2.42	2.67	2.61	2.58	0.17
8	1.78	1.59	2.10	1.34	2.29	1.82	0.38
9	2.54	1.84	1.84	1.78	1.40	1.88	0.41
10	1.46	2.10	1.46	1.34	1.14	1.50	0.36
11	1.59	1.34	1.21	1.21	1.59	1.39	0.19
12	1.53	1.72	1.65	1.84	1.40	1.63	0.17
13	1.78	1.46	1.72	1.59	1.21	1.55	0.23
14	1.46	1.21	1.53	0.95	1.34	1.30	0.23
15	0.51	1.14	1.02	1.14	1.08	0.98	0.27
16	0.83	0.95	0.57	0.64	0.83	0.76	0.16
17	1.02	0.83	0.95	1.14	0.76	0.94	0.15
18	0.89	0.95	0.70	1.08	0.64	0.85	0.18
19	1.34	0.76	1.34	1.14	0.83	1.08	0.27
20	1.02	1.08	1.02	0.83	1.21	1.03	0.14

**Table 8:** 8 instances to illuminate accurate prediction of new indications in validation test dataset.

Name	ID	1st order prediction	2nd order prediction	Original indication	New indication
Rapamycin	CID005284616	Antineoplastic^a^	Antiinflammatory^c^	Immunosuppressant (acted as mTOR inhibitor) [67]	Colorectal cancer, lymphoma, leukemia [68, 69]
Zoledronic	CID000068740	Antineoplastic^a^	Antiinflammatory^c^	Antibone resorption (acted as osteoclast inhibitor) [70]	Multiple myeloma, Prostate cancer, breast cancer [71, 72]
Wortmannin	CID000312145	Antidiabetic^b^	Antineoplastic^a^	Antifungal [25]	Leukemia [73]
Galantamine	CID000009651	Anti-Alzheimer's disease^a^	Antihypertensive^c^	Polio (acted as acetylcholinesterase inhibitor) [1]	Alzheimer's disease [1]
Ropinirole	CID000005095	Antipsychotic^c^	Antiparkinsonian^a^	Antihypertension (acted as dopamine-2 agonist) [1]	Parkinson's disease [1]
Zidovudine	CID000035370	Antiviral^b^	Antineoplastic^a^	Anticancer [1]	Anti-HIV [1]
Allopurinol	CID000002094	Uricosuric^a^	Antineoplastic^b^	Tumor lysis syndrome [74]	Gout [75]
Colesevelam	CID000160051	Antihypolipidemic^b^	Antidiabetic^a^	Antihyperlipidemia [64]	Type 2 diabetes mellitus [65, 66]

a: correctly predicted in new indications;

b: correctly predicted in original indications;

c: incorrectly predicted in original indications.
